# Pain in critically ill COVID-19 patients: An Italian retrospective study

**DOI:** 10.1515/med-2022-0600

**Published:** 2022-11-17

**Authors:** Emiliano Petrucci, Vincenza Cofini, Barbara Pizzi, Marco Cascella, Gioele Marrocco, Gianmaria Ceccaroni, Stefano Necozione, Alessandro Vittori, Franco Marinangeli

**Affiliations:** Department of Anesthesia and Intensive Care Unit, San Salvatore Academic Hospital of L’Aquila, 67100, L’Aquila, Italy; Department of Life, Health and Environmental Sciences, University of L’Aquila, 67100, L’Aquila, Italy; Department of Anesthesia and Intensive Care Unit, SS Filippo and Nicola Academic Hospital of Avezzano, 67051, Avezzano, L’Aquila, Italy; Department of Anesthesia and Critical Care, Istituto Nazionale Tumori, IRCCS, Fondazione Pascale, 80131, Naples, Italy; Department of Anesthesiology, Intensive Care and Pain Treatment, University of L’Aquila, L’Aquila, Italy; Department of Anesthesia and Critical Care, ARCO ROMA, Ospedale Pediatrico Bambino Gesù IRCCS, Piazza S. Onofrio 4, 00165 Rome, Italy

**Keywords:** pain, COVID-19, ARDS, post-intensive care syndrome, neuropathic pain

## Abstract

We retrospectively analyzed the data from patients admitted to the intensive care unit (ICU) of the Hospital of L’Aquila during the first and second waves of pandemic to identify pain related to COVID-19. Pain was evaluated by using the Numerical Rating Scale, and the assessment for neuropathic disturbances of pain was performed with von Frey’s hair and Lindblom tests. Pain increased significantly during hospitalization (from 48% at hospital admission to 94.3% at ICU discharge). Female patients were affected by somatic pain in 32.8% of the cases and by somatic pain and pain with neuropathic features (NFs) in 23.5% of the cases, during the ICU stay. Somatic pain and pain with NFs affected more frequently patients with cardiological and respiratory comorbidities. Patients treated with continuous positive airway pressure via helmet had a higher frequency of somatic pain and pain with neuropathic disturbances (84 and 74%, respectively). The frequency of somatic pain and pain with neuropathic disturbances was lower in patients sedated with propofol combined with ketamine. Females have been associated with a higher risk of somatic pain and pain with NFs. Patients with cardiological and respiratory comorbidities undergoing noninvasive ventilation had higher levels of pain. As conclusion, ketamine may reduce the promotion or the worsening of pain in COVID-19 patients.

## 1 Introduction

Post-intensive care syndrome (PICS) is a complex multifactorial condition that comprehends cognitive, physical, and psychological dysfunction reported after intensive care unit (ICU) discharge. Persistent post-intensive care (PPIC) somatic pain with neuropathic features (NFs) is often part of this syndrome. There is a potential risk of post-COVID syndrome following ICU in patients with severe acute respiratory syndrome coronavirus 2 (SARS-CoV-2) [1,2,3,4,5]. Three months after ICU discharge, critical illness polyneuropathy and myopathy syndrome with somatic and neuropathic pain has been reported in approximately 25–45% of critically ill patients after intensive care stays treated with noninvasive or invasive mechanical ventilation (IMV) [6]. Commonly administered sedative agents (SAs) and neuromuscular blocking agents (NMBAs) used for patient comfort and lung-protective ventilation have been recognized as risk factors of these syndromes [7,8].

Actually, the question whether the clinical history and pharmacological treatments, including SAs and NMBAs, can influence the development or the increase in somatic pain with NFs in patients requiring respiratory support is poorly understood [9].

Considering the lack of studies assessing persistent symptoms, including pain following ICU discharge, this study aimed to investigate somatic pain associated with NFs in PICS of SARS-CoV-2 patients admitted in ICU for respiratory support.

To accomplish this, we retrospectively reported the clinical features and treatments and their possible influence on the development or the increase in pain in SARS-Cov-2 patients requiring ICU admission for respiratory support [10].

## 2 Methods

We retrospectively analyzed the data from SARS-Cov-2 patients admitted to the ICU of the COVID Hospital of L’Aquila (Italy) in accordance with the STROBE Statement.

### 2.1 Data sources

Data were collected from electronic medical records. The information was collected from emergency department records and discharge reports. COVID-19 infection was confirmed by nasopharyngeal swabs and by real-time polymerase chain reaction assay performed by the hospital-based clinical laboratory [11]. We included all patients with severe COVID-19 disease, who were admitted to ICU between April 30th, 2020 and May 20th, 2021. Patients with consciousness impairment and patients who died were excluded.

The objective was to investigate the clinical characteristics associated with pain development in ICU admitted respiratory support SARS-Cov-2 patients.

### 2.2 Variables

Baseline variables included the following: demographic data, presence of comorbidities, and treatments administered during the permanence in ICU.

Data collected included the following: sex, age, body mass index (BMI, kg m^−2^), time spent in ICU (days), development of acute respiratory distress syndrome (ARDS, yes/no), ventilation strategy (needing continuous positive airway pressure [CPAP, yes/no] with helmet, or noninvasive ventilation [NIV, yes/no] via full-face mask [FFM] or helmet, or IMV [yes/no] via oro-tracheal tube [OTT] followed or not by tracheostomy [Ts], pronosupination [yes/no]), SA and NMBA consumption, and history of somatic pain and NFs at the moment of hospital admission (yes/no) and at ICU discharge (yes/no).

Comorbidities included cardiovascular diseases, respiratory diseases, cancer, metabolic disorders, neurological disorders, and renal disorders.

This research analyzed data collected at the time of ICU admission, during the hospitalization, and at the moment of discharge.

Somatic pain was considered as the somatic nociceptive pain (superficial and deep) arising from the skin, soft and musculoskeletal tissue, due to actual or threatened damage to non-neural tissue with activation of nociceptors. Pain was defined according to the International Association for the Study of Pain (IASP) [12].

Pain levels were evaluated according to Numerical Rating Scale (NRS, an 11-point numeric rating scale from 0 [no pain] to 10 [worst imaginable pain]). Patients with an NRS score ≥4 were considered with somatic pain.

We considered as NFs of pain the combination of sensory loss and pain either with or without sensory hypersensitivity phenomena in the somatic painful area [13].

The assessment for NFs of pain was performed by testing the skin of arms, legs, abdomen, and back using von Frey’s hair test and the Lindblom test [14,15,16].

The positivity of the tests for one of the evaluated features (allodynia, dysesthesia, hypoesthesia, and hyperesthesia) was considered a sign of neuropathic disturbance, due to a somatosensory nervous system abnormal function [17].

At the time of this analysis, our institution experienced shortages of fentanyl, sufentanil, midazolam, and dexmedetomidine. The decision about the use of SAs was led by the drug’s availability during the emergency. For this reason, our strategy for patient sedation was the continuous intravenously (IV) adjunctive infusion of ketamine (PK) or remifentanil to propofol (PR). SAs were administered at the moment of ICU admission and during the hospitalization, based on the need of respiratory support.

Patients received 3–5 mg kg^−1^ h^−1^ of IV propofol combined with 0.05–0.10 μg kg^−1^ min^−1^ of IV remifentanil or combined with 0.12 mg kg^−1^ h^−1^ of IV ketamine [18,19].

A continuous IV infusion of 0.4 mg kg^−1^ h^−1^ of rocuronium bromide was administered to patients who required OTT or underwent resupination procedure, during IMV.

The sedation strategy was titrated to achieve a moderate to deep sedation (−3 to −4) using the Richmond Agitation and Sedation Scale [20].

The respiratory support consisted of NIV procedures (CPAP with helmet or NIV via FFM or helmet) or IMV procedures via OTT followed or not by Ts. The respiratory support in patients treated with CPAP consisted of positive-end respiratory pressure (PEEP) values of 8–10 cm H_2_O, while for the NIV strategy PEEP values were 8–10 cm H_2_O with pressure support (PS) of 8–12 cm H_2_O. Patients treated with IMV received a tidal volume of 4–6 mL kg^−1^, PEEP values from 13 to 24 cm H_2_O, and respiration rates were titrated in order to maintain pulse oximetry >90% and a pH >7.2 [21]. Arterial blood gas analysis (ABGA) and lung ultrasound score (LUSS) were performed three times a day [22].

Specific pharmacological treatment with antiviral therapy, including hydroxychloroquine, lopinavir/ritonavir, remdesivir, and plasma taken from convalescent donors of COVID-19, was administered [23,24] in all patients during ICU admission and during the hospitalization, in accordance with the Italian National Health Service recommendations [25].

### 2.3 Statistical analysis

We describe qualitative and ordinal variables as frequencies and percentages, respectively, and quantitative continuous variables as mean values and standard deviations (SD). The proportion within groups was compared using Cochran’s test. The Chi-square test or the Fisher exact test was used for categorical variables, while the Kruskal–Wallis test was used to compare continuous variables among independent groups.

The level of significance was set to 0.05. Statistical analysis was performed with Stata 14.


**Informed consent:** Written informed consent was obtained from all subjects or their proxies or legal surrogates.
**Ethical approval**: This study was approved by L’Aquila and Teramo Ethics Committee (approval number: 26100/21; date of registration: 24/02/2021).

## 3 Results

During the study period, 123 patients fulfilled the inclusion criteria. As reported in Table 1, survivor patients were mostly males (71%; 87/123) with a mean age of 64 (SD: 14.7); 72 patients had more than 3 pathologies; 33 patients were treated with IMV via OTT followed by Ts. The combination of PR was administered to 52 patients (42%), while 71 patients (58%) were treated with PK (Table 1).

**Table 1:** Characteristics of COVID-19 patients at hospital admission

	*N* = 123
Characteristics	*N* (%) or median (IQR)
Gender	
Female	36 (29)
Male	87 (71)
Age (years)	67 (25)
BMI (kg m^−2^)	25 (3.2)
Diseases	
Cardiovascular (yes)	113 (92)
Metabolic (yes)	47 (38)
Respiratory (yes)	58 (47)
Neurological (yes)	28 (23)
Renal (yes)	29 (24)
Cancer (yes)	22 (18)
Other (yes)	42 (34)
ARDS (yes)	66 (54)
Time spent in ICU (days)	14 (6)
Ventilation strategy	
CPAP (yes)	26 (21)
NIV followed IMV (yes)	28 (23)
NIV (yes)	64 (52)
IMV (yes)	5 (4)
SAs	
PR	52 (42)
PK	71 (58)
NMBAs (yes)	42 (34)

Abbreviations: BMI: body mass index; ARDS: acute respiratory distress syndrome; ICU: intensive care unit; CPAP: continuous positive airway pressure; NIV: noninvasive mechanical ventilation; IMV: invasive mechanical ventilation; SAs: sedative agents; PR: combination of propofol + remifentanil; PK: combination of propofol + ketamine; NMBAs: neuromuscular blocking agents.

During admission, 59 out of 123 patients reported pain (48%; 95% CI: 39−57%), and they were classified as patients with somatic pain (36), pain with NFs (11), and patients with somatic pain and pain with NFs (12), as reported in Table 2.

**Table 2:** Patient’s classification by pain

	*n*	%
Somatic pain	36	29.27
Pain with NFs	11	8.94
Somatic pain and pain with NFs	12	9.76
No pain	64	52.03

NFs: neuropathic features.

**Figure 1:**
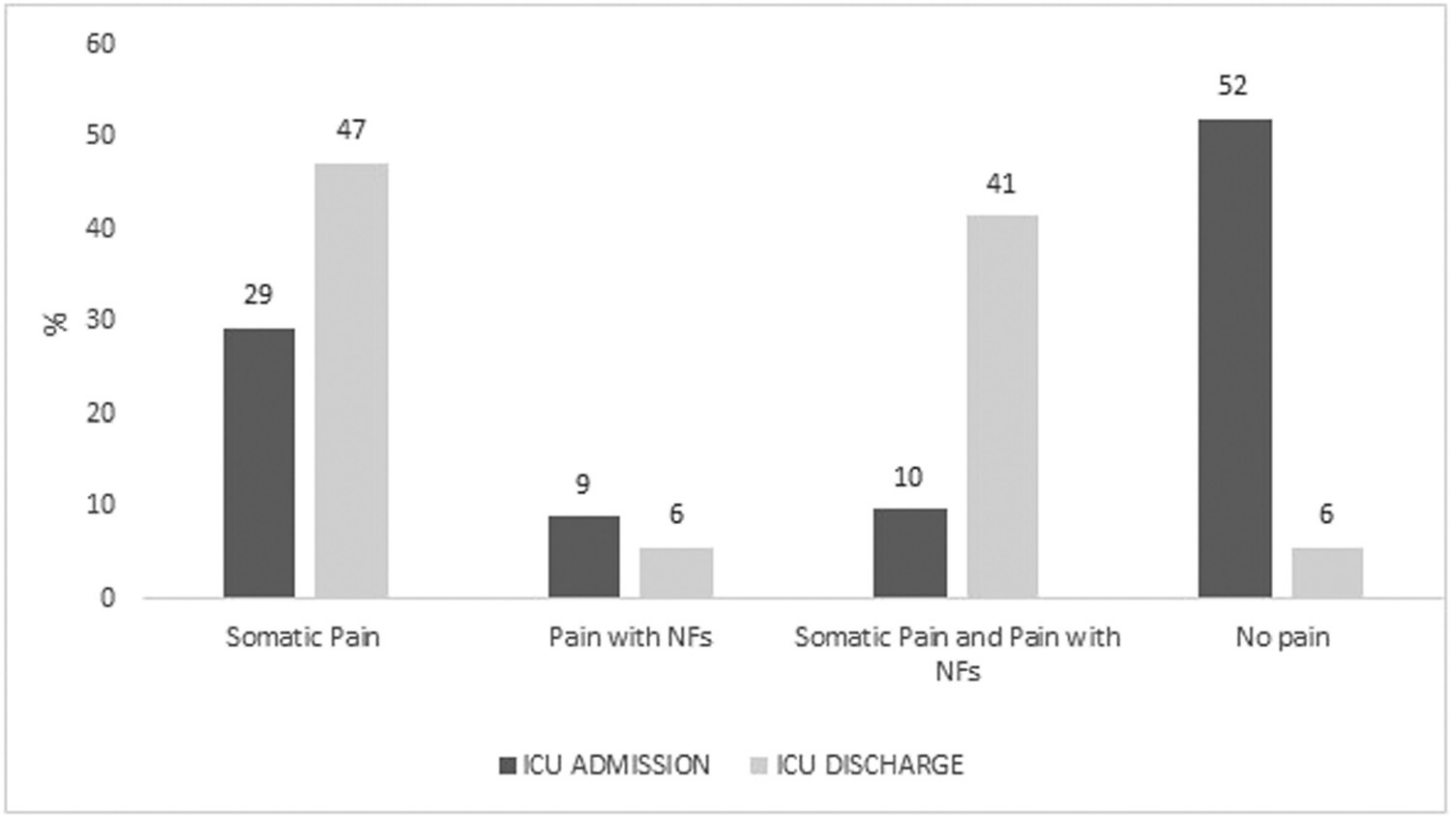
Neuropathic pain feature proportions by time.

At ICU discharge, 116 patients reported pain (94.3%; 95% CI: 88.4−97.3%), indicating a significant difference over time (Cochran’s chi2 = 51.57; *p* < 0.001). Figure 1 reports the distribution of the patients over time by pain classification: all comparisons resulted in statistically significant value (*p* < 0.05). This indicates a decrease in the proportion of patients with pain with NFs (9% vs 6%) during the permanence in ICU. Just 6% of the admitted patients were pain-free.

Table 3 reports the patient’s characteristics by type of pain. During the ICU hospitalization, male patients were affected by somatic pain at 67.2% of the cases (39 patients out of 58) and by somatic pain and pain with NFs at 76.5% of the cases (39 patients out of 51), while only 19 women (32.8%) had somatic pain. Somatic pain with NFs was recorded in 12 female patients (23.5%). Cardiological disorders and respiratory disorders were observed in 57 (93%) and in 33 (57%) patients with somatic pain, respectively. Patients with somatic pain with NFs were affected by cardiological disorders in 82% of the cases (42 patients out of 51). With respect to ventilation strategy, 84% of the patients (49) with somatic pain were treated by CPAP via helmet and 74% of the cases suffered from somatic pain and pain with NFs (38 patients out of 51). On the contrary, 9 patients treated with mechanical ventilation (invasive or noninvasive) had somatic pain (16%: 9/58) and 26% of the cases (13/51) reported somatic pain and somatic pain with NFs. All patients with pain with NFs received CPAP with a helmet, while no patients treated with mechanical ventilation had this symptom. The PK combination was administered on 42 patients (75%) who reported somatic pain, while 14 patients (25%) received the PR combination as a sedation strategy. Propofol was combined with remifentanil as SAs in 32 patients (63%) with somatic pain and pain with NFs, while 19 patients (37%) with these disturbances received the combination with ketamine. NMBAs were used in 18 patients (31%) who had somatic pain and in 17 patients (33%) suffering from somatic pain and pain with NFs.

**Table 3:** Frequency distribution of patients’ characteristics during ICU staying by perceived pain

Variable	Somatic pain (*n* = 58)	Pain with NFs (*n* = 7)	Somatic pain and pain with NFs (*n* = 51)	No pain (*n* = 7)	*p*
Female sex	19 (32.8%)	3 (42.9%)	12 (23.5%)	2 (28.6%)	0.620
Age (years)	63 (SD: 15.3)	65 (SD: 15.5)	64 (SD: 14.5)	68 (SD: 12.2)	0.8299
BMI	22.5 (SD: 2.4)	24.4 (SD: 4.4)	23.5 (SD: 3.2)	24.1 (SD: 3.1)	
Time stay (ICU)	12.3 (SD: 5.3)	17.3 (SD: 3.7)	14.0 (SD: 4.0)	13.8 (SD: 2.2)	0.0438
Cardiological disorders (yes)	57 (93%)	7 (100%)	42 (82%)	7 (100%)	0.014
Metabolic disorders (yes)	28 (48%)	4 (57%)	12 (23%)	3 (43%)	0.041
Respiratory disorders (yes)	33 (57%)	5 (71%)	17 (33%)	3 (43%)	0.050
Neurological disorders (yes)	12 (21%)	1 (14%)	11 (22%)	4 (57%)	0.159
Renal disorders (yes)	15 (26%)	0 (0%)	12 (23%)	2 (29%)	—
Cancer (yes)	2 (3%)	2 (29%)	18 (35%)	0 (0%)	—
Other disorders (yes)	20 (34%)	4 (57%)	17 (57%)	1 (33%)	0.409
Ventilation strategy					
Continuous positive airway pressure via helmet	49 (84%)	7 (100%)	38 (74%)	3 (43%)	0.029
Mechanical ventilation (invasive or noninvasive)	9 (16%)	0 (0%)	13 (26%)	4 (57%)	
SAs: sedative agents					
PR: a combination of propofol + remifentanil	14 (25%)	2 (29%)	32 (63%)	4 (57%)	
PK: a combination of propofol + ketamine	42 (75%)	5 (71%)	19 (37%)	3 (43%)	0.001
NMBAs: neuromuscular blocking agents	18 (31%)	1 (14%)	17 (33%)	1 (33%)	0.584

## 4 Discussion

We found that the distribution of the patients with pain increased significantly over time (from 48% at hospital admission to 94.3% at the ICU discharge). A significant difference (*p* < 0.05) was noted between the somatic pain and somatic pain with NFs, indicating that during the permanence in ICU, there was an increase in the proportion of patients with these symptoms. At the moment of ICU discharge, only 6% of the patients were pain free, and we observed a decrease in the number of patients with NFs of pain (9% vs 6%). These data seem to be aligned with the extensive body of literature, which clearly suggests that during ICU treatment up to 40% to 70% of the patients experience pain [26]. In addition, these results can confirm the possible existence of a PICS in SARS-CoV-2 patients who survived the acute phase of the disease post-COVID syndrome after the ICU discharge, as postulated by Vittori et al. [1].

The evidence regarding sex differences in pain perception suggests that male and female patients requiring ICU admission differ in their responses to pain. An increased pain sensitivity and risk for clinical pain is commonly observed in women, demonstrating that pain is more frequently reported by women than men [27,28]. Pain is the most common symptom during an active COVID-19 infection. We found that female patients were affected by somatic pain in 32.8% of the cases and by somatic pain and pain with NFs in 23.5% of the cases, during the ICU hospitalization. These data may be explained by assuming that women appear to be twice as likely to experience pain as men but until around age 60 years, when the risk level becomes similar [29].

We also found that somatic pain and somatic pain with NFs were more common in patient with cardiological and respiratory disease comorbidities. A significantly higher prevalence of pain in adults (aged 65 and over) who are also affected with cardiological disease has been demonstrated, but it is still not possible to clearly qualify the strength of this association [30]. Recent retrospective studies reported a higher incidence of cardiovascular disease in COVID-19 patients, underlying that cardiovascular and respiratory comorbidities may be risk factors for poor prognosis during ICU admission for respiratory support and for development of a post-COVID syndrome with PPIC somatic pain with NFs as postulated by Vittori et al. [1,2,4,31].

Our data underline that ventilation strategy may influence pain development or attribute to an increasing level of pain in critically ill SARS-Cov-2 patients. Pain in ICU patients is a complex multifactorial condition. Intubated and mechanically ventilated patients cannot fit the IASP definition of pain, as they cannot self-report pain sensations or assess their intensity [32]. Respecting these premises, patients treated with CPAP via helmet had a higher frequency of somatic pain and pain with NFs (84 and 74%, respectively) than patients who received mechanical ventilation. Sedation treatments and NMBAs administered to improve patient’s comfort and to facilitate lung-protective ventilation may have impaired the evaluation of pain in these patients. This is one of the drawbacks of this research. With respect to sedation treatments, the frequency of somatic pain and pain with NFs is lower in patients treated with PK combination than in patients who received the combination of propofol and remifentanil. We explain our results by considering the pharmacological profile of each SA. Ketamine causes bronchodilation, increase in blood pressure and heart rate by releasing endogenous catecholamines and maintaining respiratory drive and airway reflexes, without opioid-induced side effects with some benefits in promoting weaning from respiratory support, probably due to the minimal impairment of the diaphragmatic and respiratory muscular function, in comparison with the combination of PR [7,33,34].

Ketamine also has anti-inflammatory properties. Significant decrease in plasma levels of interleukin-6 (IL-6) and C-reactive protein was observed after administration of ketamine in general surgical and cardiac surgical patients, as reported by Dale et al., compared with an opioid-based sedation [35]. After ketamine IV injection tumor-necrosis factor-α (TNF-α) and TNF-α receptor 1 also remained stable, as well as leukocyte counts [19].

On the contrary, remifentanil is an ultra-short-acting µ-opioid receptor agonist, which often generates and strengthens postoperative pain sensitization, known as remifentanil-induced postoperative hyperalgesia. Evidence suggests that the transient receptor potential vanilloid 1 (TRPV1) is involved in the development of neuropathic pain and hyperalgesia [23]. Continuous IV infusion of remifentanil induced thermal hyperalgesia and mechanical allodynia, which were accompanied by upregulation of TRPV1 and protein kinase C in dorsal root ganglion. Remifentanil also increases the TNF-α, IL-1β, and IL-6 levels and activates the NMDA receptors via the activation of calcium/calmodulin-dependent protein kinase II signaling pathways in DRG neurons [28]. The NMDA receptor activation plays a central role for the development of central and peripheral sensitization, which may contribute to develop or increase somatic pain with NFs [36].

Differently from remifentanil, the immune modulation effect and the NMDA receptor signaling pathway inhibition provide prolonged continuous IV infusion of ketamine (>10 days), which may attenuate the COVID-19-induced or increased neuropathic pain, avoiding central sensitization and pain hypersensitivity [36]. Furthermore, Park et al. noted that 1 month after discharge, 10–50% of COVID-19 population reported PICS after abrupt awakening from sedation with disorientation and severe stress. This may contribute to the development or increase the somatic pain with NFs [37,38]. On the contrary, the wearing off of the pharmacological effects of ketamine is slow, due to its long-acting metabolites.

Several limitations affect this research. First, this was a retrospective observational study with all related limitations of this design. An important drawback was that the correlation between weaning time from respiratory support and SAs results from ABGA and LUSS test, NMBA consumption and specific pharmacological treatment with antiviral therapy were not studied. Furthermore, we were not able to investigate other determinant conditions (e.g., cognitive, physical, and psychological dysfunctions), which may in turn promote the development of PPIC somatic pain with NF after the ICU discharge. In addition, we were not able to evaluate the time spent in prone position by each patient and the rehabilitation time. There was also a lack of data regarding specific pharmacological pain treatments among survivors.

In conclusion, our findings confirm the presence of somatic pain with NFs as a part of PICS in SARS-CoV-2 patients admitted in ICU for respiratory support. Sex, comorbidities, ventilatory strategy, and SAs might influence the development or the increase in somatic pain and neuropathies, presumably mitigating the long-term nociceptive hypersensitivity COVID19- and opioid-related effects. We believe that findings add significant evidence to the growing literature on pain prevention and treatment as a part of PICS, after acute phase of COVID 19 infection in critically ill patients. Further studies will be needed to confirm our findings.
